# Auxin and Cytokinin Interplay during Leaf Morphogenesis and Phyllotaxy

**DOI:** 10.3390/plants10081732

**Published:** 2021-08-21

**Authors:** Sajid Hussain, Satyabrata Nanda, Junhua Zhang, Muhammad Ishaq Asif Rehmani, Muhammad Suleman, Gaojie Li, Hongwei Hou

**Affiliations:** 1 The Key Laboratory of Aquatic Biodiversity and Conservation of Chinese Academy of Sciences, Institute of Hydrobiology, Chinese Academy of Sciences, Wuhan 430072, China; hussainsajid@caas.cn; 2 State Key Laboratory of Rice Biology, China National Rice Research Institute, Hangzhou 310006, China; sbn.satyananda@gmail.com (S.N.); zhangjunhua@caas.cn (J.Z.); 3 Soil and Water Testing Laboratory, Marketing Division, Pak Arab Fertilizer Limited, Multan 66000, Pakistan; sulemanquresi@yahoo.com; 4 MS Swaminathan School of Agriculture, Centurion University of Technology and Management, Odisha 761200, India; 5 Department of Agronomy, Ghazi University, Dera Ghazi Khan 32200, Pakistan; mrehmani@gudgk.edu.pk

**Keywords:** leaf formation, leaf arrangement, phytohormonal crosstalk, plant development

## Abstract

Auxins (IAA) and cytokinins (CKs) are the most influential phytohormones, having multifaceted roles in plants. They are key regulators of plant growth and developmental processes. Additionally, their interplay exerts tight control on plant development and differentiation. Although several reviews have been published detailing the auxin-cytokinin interplay in controlling root growth and differentiation, their roles in the shoot, particularly in leaf morphogenesis are largely unexplored. Recent reports have provided new insights on the roles of these two hormones and their interplay on leaf growth and development. In this review, we focus on the effect of auxins, CKs, and their interactions in regulating leaf morphogenesis. Additionally, the regulatory effects of the auxins and CKs interplay on the phyllotaxy of plants are discussed.

## 1. Introduction

Most of the higher plants owe their evolutionary fitness partially to their continuous network of interconnected vascular cells [1]. This network of cells provides structural scaffolding for the plant organs and ensures efficient transport of the water, minerals, hormones, and photosynthesis. The highly predictable and characteristic vascular patterns are tissue-specific in many species, suggesting a genetically based development process. Plants are constantly exposed to the environment due to lack of mobility, which in turn evolved the plants with exceptional survival strategies and developmental plasticity. Plant hormones, also known as phytohormones, play crucial roles in plant development and architecture modulation. More often, phytohormone pathways are complex but fine-tuned and share cross paths with other hormonal pathways. Two such phytohormones with vital roles in plants are auxins and cytokinins (CKs), and their biosynthesis pathways are well characterized in plants [2]. Auxins and CKs regulate the key physiological processes in plants by acting both antagonistically and synergistically [3]. For instance, the development of shoot meristems by the proliferation and differentiation of meristematic cells is regulated by the antagonistic interactions of auxins and CKs [4]. In contrast, plant growth and development can be modulated by the synergistic inputs of auxins and CKs [5]. 

Moreover, the in-depth analysis of auxins and CKs pathways has revealed that multiple players are involved in the crosstalk of these two hormones, fine-tuning their levels of auxins and cytokinins in plants [6]. The cytokinins biosynthesis is positively modulated by auxins via direct control of the *ISOPENTENYL TRANSFERASE (IPT)* genes transcript accumulations, which is mediated by the *AUXIN RESPONSE FACTOR 19 (ARF19)* transcription factor [5]. Similarly, the auxin-mediated transcriptional control of *CYTOKININ RESPONSE FACTOR 2 (CRF2)* by *AUXIN RESPONSE FACTOR 5* (*ARF5*) can regulate the cytokinin pathway [7]. On the other hand, CKs manage the auxin distributions in plant cells by coordinating the influx and efflux carrier expressions. For example, transcription and translational activations of *AUXIN/LIKE AUX* (*Aux/LAX*) genes and *PIN-FORMED (PIN)* family genes are regulated by CKs [8,9]. As both auxins and CKs are crucial for cell proliferation and the development of organs in plants, characterizing their interplay becomes even more important. In this regard, the available literature with the recent findings sheds light on the involvement of both auxin and cytokinin pathway genes in leaf development in plants. However, the available literature is not sufficient for understanding the underlying molecular mechanisms [6,10]. 

In plants, the formation of leaves is a continuous developmental process [11]. Leaves are crucial plant organs having highly organized functions, such as photosynthesis and respiration. In addition, the leaf structures and arrangements vary within and among plant species. It is confirmed that auxins contribute towards leaf development, including leaf initiation, blade development, and compound leaf patterning [12]. Similarly, auxin and CKs play important roles in many aspects of plant growth and development; particularly in the formation of meristem cells, which are vital to establishing the whole plant body [13]. CKs control several key developmental functions in plants, such as the regulation of shoot apical meristem (SAM) to leaf senescence. Further, the SAM is formed during the embryogenesis process, from which almost all of the aerial parts of a plant are developed. Cell division and cell differentiation actively occur in SAM. SAM is subdivided into various regions such as central, peripheral, and rib zones. Among these zones, the peripheral zone of the SAM is responsible for leaf primordial origination, which is replaced by cell division in both the peripheral zone itself and the central zone [13,14,15].

To elucidate this complex role of cytokinin in leaf development, temporal CK levels were investigated in the leaf of *Arabidopsis*. At the cell proliferation phase, CKs are needed to maintain cell proliferation by blocking the transition to cell expansion and the onset of photosynthesis. As a result, a CK excess at the cell expansion phase results in the increased leaf and rosette size, enhanced by a higher cell expansion rate, yielding higher shoot biomass. Further, the proteome profiling studies revealed that the stimulation of primary metabolism by CK in line with an increased sugar content increased the turgor pressure, representing the driving force of cell expansion. Therefore, the developmental timing of CK content fluctuations, together with tight control of primary metabolism, is a key factor mediating transitions from cell proliferation to cell expansion in leaves [16]. However, the precise mechanism behind vein pattern formation in plant leaves is not well known.

In this review, we discuss how auxins and CKs play roles in controlling the development and differentiation of leaves. We summarize recent progress in understanding how auxins and CKs signaling and their interactions regulate leaf morphogenesis. Finally, we have proposed a conceptual model depicting the auxins and CKs signal-mediated regulation of the leaf development.

## 2. General Leaf Development Mechanism

In leaves, the vascular tissue is derived from elongated precursor cells called procambium, which is formed from the undifferentiated ground meristem (GM) cell population and later differentiates into vascular elements, i.e., xylem and phloem [17]. During this process, the leaf is expanding through cell divisions, which slows down and stops first in the distal, and then, in the proximal part of the leaf [18]. This results in the formation of larger vascular bundles in the proximal regions of the leaf, even along the same strand [19]. Strands are often categorized into three vein orders: the primary veins (the midvein) are considered as the linear procambium or vascular strand approximately along the midline, secondary veins are those vascular strands (procambium) connected to the midvein, and tertiary veins are connected to secondary vein but not the midvein, while quaternary veins are connected to tertiary veins but not the mid-vein nor secondary veins.

To better understand the development of procambium, several vascular-specific reporter genes have been examined in the developmental series. The *ET1335::GUS* and *AtHB8::GUS* expression lines are reliable markers of procambium and both pre-procambium and procambium, respectively. They have revealed the patterns in the procambium development and differentiation in the *Arabidopsis* first leaf [17]. Based on *AtHB-8* expression, the pre-procambia secondaries develop acropetally, that is, from the mid-vein to the tip. Based on the procambium cell diagnostic traits (i.e., cell elongation), pre-procambial secondaries differentiate into procambium either rapidly or simultaneously relative to all other cells of the same strand [17]. The fact that strands of *AtHB8* include either procambium or GM but never both in the same strand supports this conclusion [17].

In the first leaf, the formation of the vein orders follows a relatively consistent pattern based on the use of the above-mentioned marker lines and the appearance of the elongated procambium [17]. Between day 2 and day 2.5 (from germination), the leaf primordium lacks any sign of procambial cells. On day 3, procambium appears along the midline, forming the presumptive midvein. As the leaf continues to elongate, these procambial cells continue to divide [19,20], thus ensuring vascular continuity in the growing organ. The first two secondary veins appear on day 5 as arches that join at the tip of the midline procambium, forming presumptive areoles (intercostal areas). On day 5.5, two additional arches of secondary procambia appear, forming presumptive areoles below the previous two. On day 7, the primordium is 0.7 mm long. On day 8, an additional pair of secondaries appears in the most proximal region. Tertiary procambium is first seen on day 7 within the central areoles and appears in the more newly formed proximal areoles at day 8. Similar observations have been made by Baima et al. [21].

In all vein orders, differentiation into the xylem can be easily seen by lignification approximately two days after procambia strand formation [17] and generally occurs in the meristematic zones [19]. By day 21, all growth and vascular differentiation of the two first node leaves have been completed [22]. Subsequent rosette leaves are larger and have an increasing amount of vasculature. However, the venation density and the number of branch points per unit area remain relatively constant.

## 3. Auxin and Cytokinin Biosynthesis: Convergence and Divergence

### 3.1. Auxin Biosynthesis in Plant

**Figure 1:**
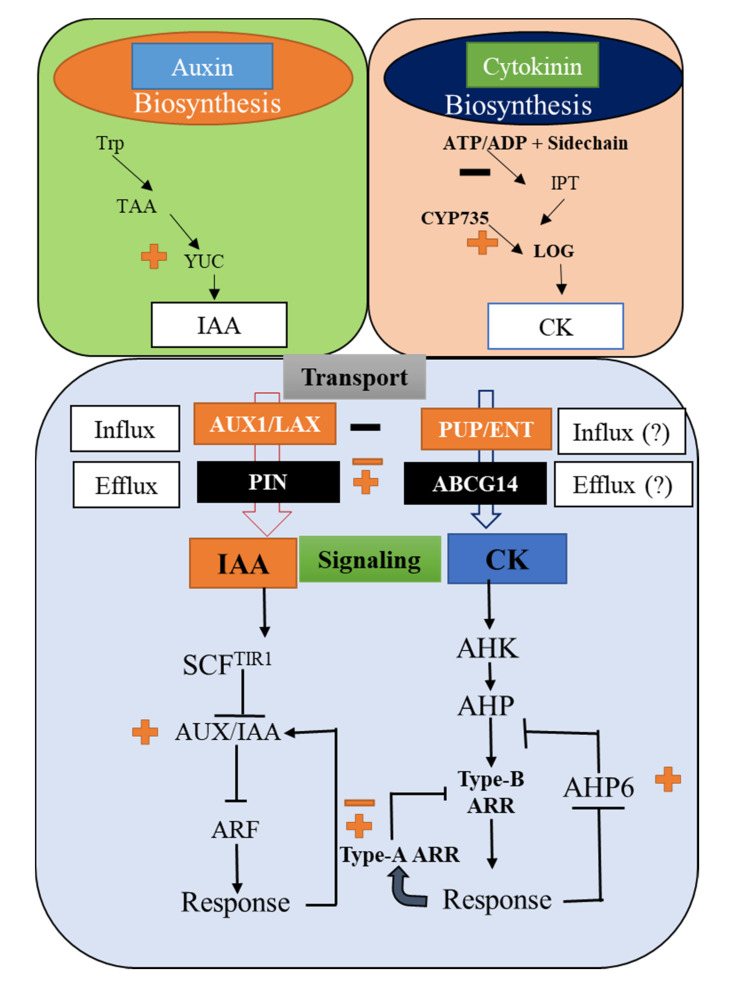
A schematic diagram depicting the biosynthesis, transport, and signaling of auxin and cytokinin in plants. Solid lines represent the established regulatory connections, whereas the dashed line indicates the possible connection. Line ending with a crossed line indicates the negative regulations.

Indole-3-acetic acid (IAA) plays a key role in plants and is mainly synthesized from the amino acid tryptophan (Trp). The first established complete Trp-dependent auxin biosynthesis pathway has two chemical steps which are essential in the plant kingdom (Figure 1). The first step is the removal of the amino group from Trp by the *Tryptophan Aminotransferase of ARABIDOPSIS* (*TAA*) family of transmission to generate indol-3-pyruvate acid (IPA). The second step is the conversion to produce IAA by *YUCCA* (*YUC*) family [23].

However, very little is known about auxin biosynthesis in plants due to its complexity [24]. With the help of genetic and biochemical approaches, new auxin biosynthesis pathways have been identified. IAA biosynthesis depends on many pathways; IAA can be released by cleavage of IAA conjugates: IAA-amino acids, IAA-methyl ester, and IAA-sugar hydrolytic. Moreover, plant species vary in nature to produce or optimize their IAA biosynthesis. IAA biosynthesis occurs through Trp by the IPA in microbes. This IPA pathway uses similar enzymes to the IPA pathway in plants. The IAA biosynthesis is also influenced by various developmental and environmental conditions [25,26]. For instance, changes in the light quality received by plants can trigger a series of developmental responses known as shade avoidance syndrome. During shade avoidance, IPA is rapidly synthesized from Trp to ensure elevated auxin biosynthesis [26]. Similarly, a local auxin biosynthesis source can modulate the auxin redistribution and gradient homeostasis, and thus, can regulate the cellular morphogenesis [25]. 

### 3.2. Cytokinin Biosynthesis in Plant

CKs were named after their biological function, e.g., cytokinesis or cell division. Later, it was discovered that cytokinins are involved in many other plant physiological processes apart from cell division [27]. In CK biosynthesis, the first step is catalyzed by the ISOPENTENYLTRANSFERASE (IPT) enzyme that adds a prenyl moiety onto ATP/ADP forming the N6-isopentenyladenine (iP) ribosides [28]. The newly formed iP ribosides can subsequently be converted to trans-zeatin (tZ)-type cytokinins by the cytochrome P450 enzymes (CYP735A1/CYP735A2) via the hydroxylation of the isoprenoid side chains [29]. Further, the LONELY GUY (LOG) family enzymes catalyze the conversion of cytokinin ribosides into free and active forms of CKs [30]. Much like auxin, the CK levels can also be decreased via conjugation, mostly with glucose [31]. The glucosyl conjugates of CKs are inactive, which can either be reversible or irreversible [32,33]. In addition, enzymatic cleavage of CKs to form tZ- and iP-type CKs can be another type of regulation to reduce the CK concentrations in plants [34,35]. CKs also display differential spatial and temporal expression and localization, giving rise to CK asymmetries that in turn regulate cellular differentiation and development [36,37]. 

On the other hand, plant CK signaling elements are encoded by a small gene family with overlapping functions [38,39]. Three well-studied cytokinin receptors are the ARABIDOPSIS HISTIDINE KINASE 2 (AHK2), AHK3, and CRE1/WOL/AHK4, containing multiple conserved domains, including a cytokinin binding CHASE domain, a histidine kinase domain, and a receiver domain [40,41]. The plant CK receptors are mostly located in the endoplasmic reticulum lumen, suggesting it to be the primary site of CK binding [42,43]. Although the key players facilitating the cellular transport of CK are not yet fully explored, some of them, including the ATP binding cassette transporters, nucleoside transporter proteins, and purine permeases have been identified to be involved in CK transport [44,45,46]. Recent reports have revealed the participation of new players in the CK signaling contributing towards the plant developments, including leaf morphogenesis. Moreover, the complex crosstalk in between auxins and CKs plays a crucial role in determining the fate of the different organ developments in the plant, including leaf formation (Figure 1). 

## 4. Effects of Auxins and CKs in Regulating Their Signaling Pathways: An Inspection

Auxins and CKs show complex interplays in plants and control various physiological activities. However, both these hormones can regulate the metabolism of one another in plants. Auxins have significant effects on CK metabolism and can exert control over its synthesis, transport, and action. For instance, the *IPT* gene expressions were influenced by the auxin levels in plants [47]. Later, it was discovered that the upregulation of *IPT* genes by higher cellular auxin levels was mediated by *SHORT HYPOCOTYL 2/INDOE-3-ACETIC ACID 3 (SHY2/IAA3)* [48]. Further, the action of auxins on *IPT* gene expressions was confirmed in plants, revealing the role of the shoot apex auxin in repressing these genes [49]. On the other hand, the treatment of auxins resulted in reduced CK biosynthesis in *Arabidopsis* [29,50].

Apart from regulating the CK biosynthesis, auxins can control its degradation by modulating the *CYTOKININ OXIDASE/DEHYDROGENASE (CKX)* gene family. In *Arabidopsis*, application of auxin down-regulated the expression of *CKX2, CKX4,* and *CKX7*, whereas the treatment of 1-naphthylphthalamic acid (NPA), a polar transport inhibitor, strongly down-regulated *CKX1* and *CKX6* expressions [51]. In addition, auxin is reported to activate *ARABIDOPSIS RESPONSE REGULATOR 7 (ARR7)* and *ARR15*, which act as CK signaling inhibitors [52]. This report suggests the direct role of auxin acting antagonistically with plant CK signaling. The *ARR7* and *ARR15* expression in *Arabidopsis* corresponded more with the auxins, not CK signaling domain, which was later confirmed by the treatment of a synthetic auxin that resulted in the ARR domain expansion. Furthermore, treatment of naphthaleneacetic acid (NAA) onto the SAM resulted in the decreased expression of *ARR7* and *ARR15*. Contrastingly, in the *arf5/mp* mutant lines, the expressions of these genes were upregulated, suggesting the auxin-dependent regulation of the ARRs mediated by *ARF5/MP* (*AUXIN RESPONSE FACTOR 5*/*MONOPTEROS*) [4]. Similarly, *CRF2*, another response regulator in the auxins-CKs interplay, was identified to be a target of *ARF5/MP* via microarray and ChIP (Chromatin Immunoprecipitation) analysis [7]. 

On the other hand, several reports confirmed that CKs could regulate auxin biosynthesis and cellular transport. For instance, the exogenous treatment of CKs onto *Arabidopsis* plants resulted in the auxin accumulations in young leaves and shoot apex. This was further validated in the *ARR* quadruple mutant (*arr3 arr4 arr5 arr6*) *Arabidopsis* lines, where the auxin biosynthesis was increased in many folds by the treatment of CKs [53]. Conversely, cytokinin treatment resulted in a significant decrease in IAA levels in the *ARABIDOPSIS THALIANA HISTIDINE PHOSPHOTRANSFER PROTEIN (AHP)* quadruple mutant (*ahp1 ahp2 ahp3 ahp4*) *Arabidopsis* plants [54,55]. IAA modulates the leaf veins development in *Arabidopsis* along with PIN1 auxin flux protein [56]. Similarly, the application of CKs resulted in the decreased expression levels of *PIN1-4* and *PIN7* in the shoot. Further, CKs treatment on pea plants caused the rapid transcript accumulation of two key players in the auxin transport, including *PsPIN1* and *PsAUX1* [57]. Getting some deeper insights into the auxins-CKs interplay, Zhang et al. [58] reported that CK-mediated regulation of auxin metabolisms, including the expression of the *PIN* class auxin transporters, might occur at a post-transcriptional level. Similar results were also concluded when CK treatments resulted in the rapid decrease in the expression levels of *PIN1* [58]. Additionally, the CK-mediated degradation of PIN1 was found to be robust in the single or double mutants of *ahk2* and *ahk3*, but compromised in the *cre/ahk4* mutant [57]. Moreover, these findings suggest that the crosstalk between auxins and CKs is complex and regulate several key physiological processes, including plant growth and development (Figure 1, Table 1). 

## 5. Role of Auxins-CKs Interactions on Leaf Development

**Figure 2:**
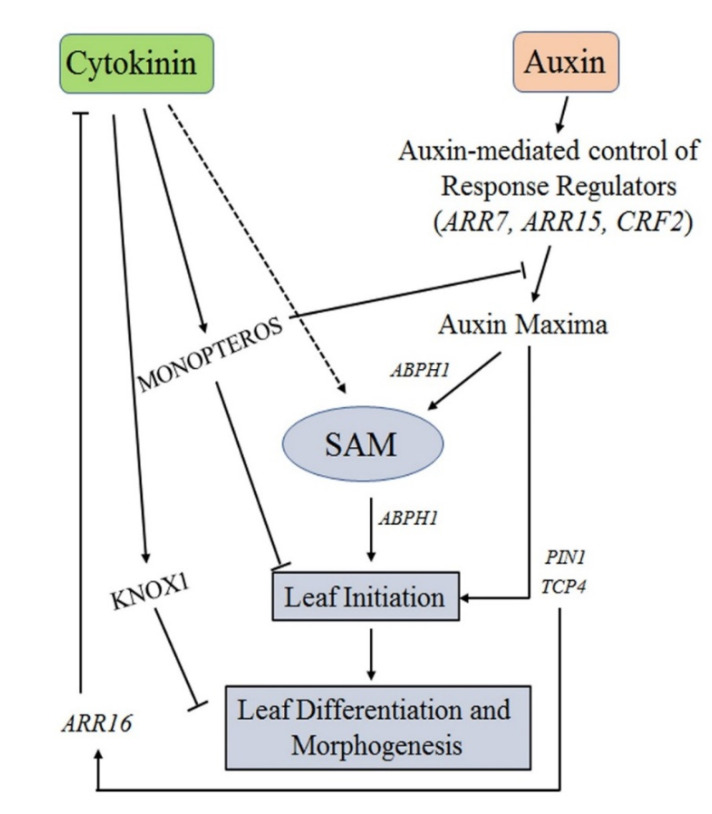
A model detailing the auxin and cytokinin interplay during leaf development and morphogenesis. Solid lines represent the established regulatory connections, whereas the dashed line indicates the possible connection. Line ending with a crossed line indicates the negative regulations.

Both auxins and CKs play a crucial role in the plant leaf morphogenesis process. While regions of auxin response maxima are generated at the SAM before the leaf initiation, CKs govern the SAM maintenance [63,64]. The leaf initiation and the whole leaf morphogenesis process banks upon a complex yet delicate balance between auxins and CKs. The CK-induced expression of *PIN1* and the response regulator protein *ABPH1* were found at the leaf initiation site in maize plants [64]. Further, the auxins-CKs interplay controls the SAM development, and phyllotaxy is highly induced by CKs and acts as a negative regulator of CK response [65]. Moreover, the possible synergistic and antagonistic nature of the auxins-CKs interactions plays a crucial role in determining the leaf initiation and development. For instance, *ARR7* and *ARR15* were negatively modulated by *MONOPTEROS (MP*) in *Arabidopsis*, whereas the *mp* mutant lines with higher cytokinin concentrations rescued the floral initiation defect [65]. Interestingly, these results suggested that auxins and CKs interactions in *Arabidopsis* show synergism for the organ initiation process in the SAM while displaying antagonism in the roots [4]. In addition, the response regulator genes, such as *ARR7* and *ARR15*, are involved in the positive control of SAM development and organ initiation in the vegetative meristems while exhibiting contrasting interactions with auxin in the meristem tissues. Similarly, *AHP6* negatively regulates CK signaling and positively controls the *Arabidopsis* floral initiation downstream of auxin [66]. Although numerous reports suggest the positive role of auxin in controlling the leaf initiation, the role of CK is more complex depending on the plant species, developmental stage, and demands in-depth research to infer any firm conclusion [63] (Figure 2). 

Beyond leaf initiation, both auxins and CKs exert significant control in leaf serration and morphogenesis [63,67]. Auxins have been reported to regulate the leaf serration and leaflet formation and separation from the compound leaf primordia margins. The inhibition of auxin signaling or transport resulted in the development of simple leaves instead of compound leaves in *Cardamine hirsuta* [63]. Furthermore, several studies have confirmed the positive role of discrete auxin maxima in promoting leaflet initiation and growth [63,68,69,70]. The localization of PIN1 at leaflet initiation site, and the exogenous auxin treatments resulting in the ectopic leaflet initiation and lamina growth, further supported the positive role of auxin maxima in leaflet initiation and growth [70]. In contrast, CKs also influences the leaf morphogenesis and differentiation balance. For instance, accelerated cell expansion and premature cell proliferation termination were realized in the leaf primordia of *Arabidopsis* by increasing the CK degradation [71]. This suggests that CKs are involved in the delaying of the cell differentiation process in the leaf primordia. CKs were discovered to act downstream of the *KNOTTEDLIKE HOMEOBOX (KNOX1)* transcription factors in delaying the leaf maturation process [72,73]. *KNOX1* genes are responsible for stem cell maintenance in the SAM and leaf development by regulating CKs and gibberellin (GA) [74,75]. It was reported that the *KNOX1-CK/GA* module also plays important role in the regulation of heterophylly (a typical representative of leaf plasticity) in many aquatic plants, such as *Hygrophila difformis* [76,77,78] and *Rorippa aquatica* [79,80]. More recently, an *Arabidopsis* transcription factor, *CYTOKININ-RESPONSIVE GROWTH REGULATOR (CKG)*, was discovered to regulate the CK-dependent cell expansion and organ growth [81]. 

On the contrary, reducing the CK leaf sensitivity in *Arabidopsis*, the *CIN (CINNATA)-TCP* transcription factor-mediated leaf maturation process was accelerated. Further, *CIN* can bind to the specific gene or promoter regions and induce the transcription of CK receptors and *SHY2/IAA3*. CIN actively participates in the auxin-CKs interplay in the leaf development by limiting the excessive cell proliferation process and ensure a flat-leaf surface. Additionally, CIN contributes to controlling the hormonal cross talks involved in cell proliferation and patterning in leaf morphogenesis [82]. Further, TCP4 induced the expression of *ARR16* that encodes a CK response inhibitor [16]. Additionally, the prolonged morphogenetic activity of a tomato leaf was maintained and regulated by CKs [79]. Recently, Muszynski et al. [83] reported that mutation in *Hairy Sheath Frayed1* (*Hsf1*), a gene regulating the leaf morphogenesis in maize, can alter leaf patterning via elevated CK signaling. Exogenous treatment of CKs onto the wild-type seedlings phenocopied the *hsf1* leaf phenotypes. The above discussion shows that the interactions between auxins and CKs can determine the shoot and leaf developments. Moreover, auxins in lower concentrations tend to limit the CK-mediated physiological processes in plants [12]. Conversely, a surge in the CK levels can counteract the inhibitory effects of auxins and negatively regulate auxin signaling. Interestingly, at higher auxin and CK levels, the antagonism between these phytohormones is significantly suppressed. Moreover, the interactions between auxins and CKs are bidirectional, as both of them tend to control each other signaling cascades [13,64]. Conclusively, the complex yet fine-tuned interplay of auxins and CKs control the leaf initiation, proliferation, and morphogenesis process. Furthermore, other phytohormones and the auxins and CKs homeostasis play important roles in determining leaf development and differentiation.

## 6. Effects of Auxin-Cytokinin Interplay in Phyllotaxy

Phyllotaxy is the way of arrangement of leaves on a plant. Both auxins and CKs have significant control over the phyllotaxy in plants. A delicate balance between cell proliferation and differentiation is maintained in the SAM, which controls the shoot development in plants [84]. CKs play a vital role in the establishment and maintenance of the central zone cell division and proliferation. Once the cell differentiation is transferred from the central zone to the peripheral zone, the organ primordia (leaf primordia) can be formed [85]. This organogenesis and its subsequent differentiation into leaves form based on phyllotaxy [86]. Conversely, both organogenesis and phyllotaxy are tightly regulated by auxins. For instance, loss of function in CK signaling can alter SAM size, whereas the auxin signaling mutation can have huge effects on organogenesis [2]. Moreover, auxin flux in the SAM area significantly affects cell patterning. Both influx and efflux auxin carriers, such as AUX/LAX and PIN families, respectively participate in this patterning of SAM [87]. The auxin levels in the cell periphery are increased by the action of AUX/LAX carriers, while the polarly localized PIN proteins create an auxin-maximum at which the primordia initiation takes place [88]. AUX proteins can be poly-ubiquitinated by SCF^TIRI^ complex, degraded through the 26S proteasome proteolytic pathway and ARF proteins were then released to trigger changes in their target auxin response genes (Figure 1). At this site, the differential localization of PIN proteins creates auxin sinks, ensuring that additional primordial is not generated in the near vicinity [89]. The effective repositioning of the auxin maxima and the auxin sink establishes the phyllotaxy around SAM, ultimately controlling the leaf arrangements in plants [89]. 

The auxins-CKs interplay exerts dynamic controls in regulating the phyllotaxy. For example, CK mutants in *Arabidopsis* exhibited an altered meristem size and abnormal phyllotaxy [90]. Likewise, rice plants carrying a CK mutation produced an enlarged shoot meristem with altered phyllotaxy [91]. Interestingly, another family of genes known as *ERECTA* can act as a buffer in regulating the effects on SAM and phyllotaxy during the auxins and CKs interplay [92,93]. Moreover, several functional genomics studies have reported the critical effects of auxins-CKs crosstalk in regulating the plant phyllotaxy. For instance, loss of function of *ABPH1* resulted in the alternation of leaf phyllotaxy and yielded in the leaf initiations in opposite pairs rather than the alternative leaf structure in maize [92]. However, a detailed analysis of the *abph1* maize plants revealed that they contained decreased levels of both auxin and *PIN1*, suggesting that the phyllotaxy alternation might have been due to the combined effect of enlarged SAM and delayed leaf initiation. Similarly, mutating the negative modulator of CK *AHP6* in *Arabidopsis* resulted in the altered phyllotaxis [85]. Interestingly, in the *ahp6* lines, no enlargement of the meristem was seen, indicating that the control of leaf arrangements via auxins-CKs interplay is more complex and tightly regulated. Moreover, in-depth analysis in the *ahp6* plants revealed that AHP6 regulates the spatiotemporal pattern of CKs at the peripheral regions of SAM, where depletion of CKs levels, in turn, elevates the auxin levels facilitating the proper phyllotactic patterning. 

## 7. Conclusions and Future Perspectives

Auxins and CKs are two of the crucial phytohormones attributed to various key physiological processes. Their individual and synergistic or antagonistic roles are well-explored in plants concerning plant growth and development. In addition, their crosstalk in regulating root development and vascular differentiation has been widely studied. However, there are only a handful of reports describing their effects in leaf morphogenesis. In this review, we have given a brief presentation on the role of auxin-cytokinin interplay in regulating the leaf morphogenesis process. From the discussion, both auxins and CKs exert tight control, independently and in association with each other, to regulate cell proliferation, cell expansion, and the transition between these processes during leaf development. Moreover, the fine-tune control of this complex interplay can efficiently regulate the leaf morphogenesis, differentiation, and arrangement in plants. Additionally, some key players, including the kinases like MAPKs and other regulatory factors, such as the non-coding RNAs in influencing the auxin-cytokinin interplay, are not fully understood during the leaf morphogenesis. Thus, in-depth analysis and functional genomics aided validations of this complex yet crucial interplay that could shed more light on the leaf morphogenesis in plants. Recently, the role of calcium ions (Ca^2+^) in Phyllotaxy regulation has been unearthed [93]. The control of Ca^2+^ flux in cells and the Ca^2+^-mediated regulation of PIN proteins have provided new insights into auxin signaling. Finally, exploring the effects of other phytohormones in regulating the auxins-CKs interplay during leaf development can help us to better understand this complex process. 

Recent advancements and discoveries on the roles of both auxins, CKs, and their cross talk in plant development have enabled us to understand the intertwined relationship between them. Further, the antagonistic and synergistic interactions of auxins and CKs in plants are now unearthed at a molecular and genetic level. With the discovery of new molecular players in the auxins and CKs interplay, the number of interaction points is also increasing in the interplay, thus making it more complex. To reduce this complexity and get a clearer picture of the auxins-CKs interplay, the role of computational biology and in silico simulation works could be of high value. Modeling the interactions and dynamics during the auxins-CKs interplay can provide new insights into the metabolisms, signaling, and transport of both hormones. However, many questions will remain unanswered without the wet-lab validation, such as what could be the principal role of cytokinin in plants concerning plant morphogenesis? Auxins have a central role in phytohormones crosstalk and plant development, and CKs actions in plants are more often coupled with auxin gradient. Could it be summarized that CKs function in plants in an auxin-dependent manner? In addition, how can auxins or CKs or their interplay contribute to the phenomenon of heterophylly and stress-associated phenotypic plasticity in plants? Moreover, delineating the complexities of the auxins-CKs interplay can provide satisfactory answers to these points. In the future, auxins and CKs can play an important role in heterophylly under salinity stress caused by osmotic stress.

## Figures and Tables

**Table 1:** Interactions of signaling pathway genes regulated by auxins and CKs.

Gene Name	Biological Function	Regulation Effect	Reference
Auxin Signaling Pathway Genes Regulated by CKs			
*AUX1*	Auxin influx transport	Upregulation	[57]
*PIN1, PIN2, PIN3, PIN4, PIN6, PIN7*	Auxin efflux transport	Lateralization, Upregulation, Tissue-specific downregulation	[54,59,60]
*IAA7, IAA13, IAA17, AMI1*	Auxin signaling	Downregulation	[61]
*AUXIN RESISTANT 1, 31 (AXR1, AXR3)*	Auxin signaling	Upregulation	[53,62]
*CYP79B2*	Auxin biosynthesis	Upregulation	[53]
*YUCCA5, YUCCA6*	Auxin biosynthesis	Temporal expression regulation	[53]
*ANTHRANILATE SYNTHASE α1 (ASA1/WEI2)*	Auxin precursor synthesis	Up-regulation	[53]
*GH3.17, GH3.9*	Auxin conjugation	Up-regulation	[53]
*PHOSPHORIBOSYLANTHRANILATE TRANSFERASE1 (PAT1)/TRYPTOPHAN BIOSYNTHESIS1 (TRP1), INDOLE-3-GLYCEROL PHOSPHATE SYNTHASE* *(IGPS), NITRILASE 3 (NIT3)*	Auxin biosynthesis	Up-regulation	[53]
CK Signaling Pathways Genes Regulated by Auxin			
*ARR7, ARR15*	Cytokinin signaling	Spatial expression regulation	[4,52]
*IPT1, IPT2*	Cytokinin biosynthesis	Downregulation	[49]
*IPT5, IPT7*	Cytokinin biosynthesis	Up-regulation	[47]
*CRF2, AHP6*	Cytokinin signaling	Up-regulation	[7,60]
*CKX2, CKX4, CKX7*	Cytokinin degradation	Downregulation	[51]
*CYP735A*	Cytokinin biosynthesis	Downregulation	[29]

## Abbreviations

*ANTHRANILATE SYNTHASE α1*	*ASA1*
*ARABIDOPSIS HISTIDINE KINASE 2*	*AHK2*
*ARABIDOPSIS HISTIDINE PHOSPHOTRANSFERASE PROTEINS*	*AHP*
*ARABIDOPSIS RESPONSE REGULATOR 7*	*ARR7*
*AUXIN RESPONSE FACTOR 19*	*ARF19*
*AUXIN/LIKE AUX*	*AUX/LAX*
*AUXIN RESISTANT 1, 3*	*AXR1, AXR3*
Chromatin Immunoprecipitation	CHIP
*class 1 KNOTTEDLIKE HOMEOBOX*	*KNOX1*
*CYTOCHROME P450 MONOOXYGENASE, FAMILY 735, SUBFAMILY A*	*CYP735A*
*CYTOKININ OXIDASE/DEHYDROGENASE*	*CKX*
CYTOKININ RESPONSE FACTOR 2	CRF2
Cytokinins	CKs
Gibberellin	GA
*GRETCHEN HAGEN 3.17, 3.9*	*GH3.17, H3.9*
Ground meristem	GM
*INDOLE-3-GLYCEROL PHOSPHATE SYNTHASE*	*IGPS*
Indole-3-pyruvate acid	IPA
Indole-3-acetic acid	IAA
ISOPENTENYLTRANSFERASE	IPT
LONELY GUY	LOG
*MONOPTEROS*	*MP*
1-naphthylphthalamic acid	NPA
N6-isopentenyladenine	iP
Naphthaleneacetic acid	NAA
*NITRILASE3*	*NIT3*
*PHOSPHORIBOSYLANTHRANILATE TRANSFERASE1*	*PAT1*
*PIN-FORMED*	*PIN*
Shoot apical meristem	SAM
*SHORT HYPOCOTYL2/Indole-3-acetic acid3*	*SHY2/IAA3*
trans-zeatin	tZ
tryptophan	Trp
*TRYPTOPHAN AMINOTRANFERASE OF ARABIDOPSIS*	*TAA*
*YUCCA*	*YUC*
